# Variation of glucose time in range in type 1 diabetes

**DOI:** 10.1002/edm2.379

**Published:** 2022-09-29

**Authors:** Klavs Würgler Hansen, Bo Martin Bibby

**Affiliations:** ^1^ Diagnostic Centre Silkeborg Regional Hospital Silkeborg Denmark; ^2^ Department of Clinical Medicine Aarhus University Aarhus Denmark; ^3^ Section for Biostatistics Department of Public Health, Aarhus University Aarhus Denmark

**Keywords:** continuous glucose monitoring, time in range, type 1 diabetes

## Abstract

**Introduction:**

The aim of the study was to assess the variation of glucose time in range (TIR) for persons with type 1 diabetes who perform intermittently scanned continuous glucose monitoring (isCGM).

**Methods:**

Glucose data for 8 weeks were analysed for 166 persons. TIR was calculated over four consecutive 2 weeks periods. Sixty‐one of the persons had two downloads with an interval of >3 months.

**Results:**

A total of 140 individuals (84%) used multiple daily injection, and 26 (16%) used continuous insulin infusion. The within‐individual standard deviation (SD) for TIR was 6.3% corresponding to 95% limits of agreement for the difference between two TIR values of ±17.6%. Mean TIR calculated from the first and last 2 weeks was 52.2 ± 17.1% and 53.7 ± 16.4%, respectively (difference 1.5%, SD of the difference 10.4%, *p* = .07). For persons with two downloads separated by months, the SD of the difference in TIR was 12.6%.

**Conclusions:**

The 95% limit of agreement for TIR is vast for persons using isCGM. It is difficult to draw firm conclusions regarding systematic differences when individual TIR from 2 weeks are compared. This may not be valid for users of insulin pumps with closed‐loop insulin delivery.

## Graphical abstract

The variation of TIR in patients who perform intermittently scanned continuous glucose monitoring (isCGM) is unknown. We report a huge 95 % limit of agreement for the difference between two TIR values. Changes in TIR values must be interpreted cautiously.

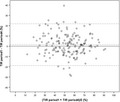

## 1 INTRODUCTION

Continuous glycaemic monitoring (CGM) has given rise to several new glycaemic metrics as valuable alternatives to haemoglobin A1c.^1^, ^2^, ^3^, ^4^ The time in range (TIR) of glucose values is strongly correlated with mean glucose and HbA1c,^5^, ^6^, ^7^, ^8^ but TIR also reflects diurnal glucose variation and is independent of individual physiological factors influencing the rate of glycation.^9^, ^10^ Another advantage of TIR is that the effect of intervention can be evaluated after a few weeks, while only minor changes in HbA1c can be expected.^8^, ^11^ It is common clinical practice to present TIR and other glycaemic indices in a one‐page condensed ambulatory glucose profile based on 14 days of CGM.^2^, ^4^ Information about spontaneous changes in TIR is sparse. This information is necessary to determine whether an observed clinically relevant change in TIR can be considered statistically significant or within the range of normal variation. TIR is an important outcome measure in clinical trials, and study dimensioning depends on information about the reproducibility of TIR.

Intermittently scanned continuous glucose monitoring (isCGM) is the most widespread form of CGM. The aim of the present study was to describe variation in TIR and other glycaemic metrics in persons with type 1 diabetes using isCGM.

## 2 MATERIAL AND METHODS

We had unrestricted access to isCGM (Freestyle Libre, Abbott) in the diabetes outpatient clinic for adults with type 1 diabetes in Regional Hospital Silkeborg, Denmark.^12^ Glucose data were evaluated from all available downloads to the Diasend platform in the period February to November 2019. The study population comprises 169 non‐pregnant individuals with type 1 diabetes of whom 61 had two downloads with an interval of more than 3 months. Glycaemic metrics from this cohort have previously been described in detail.^8^

Glycaemic metrics were evaluated for periods of 2 weeks for the last 8 weeks before download. Period 1 was weeks one and two before download and period 4 was weeks seven and eight before download. For persons with two downloads, glycaemic metrics for the last 14 days from the first and second downloads were compared.

Mean glucose was calculated as the mean of glucose values (scanned and imported) for each 15 min. Period. Active CGM time (%) was calculated for 14 days as the number of 15 min. Periods with at least one glucose value divided by the total number of 15 min. Periods (1344 = 14 × 24 × 4) multiplied by 100. TIR within a 14 days' period was the number of 15 min. Periods with a mean glucose value 3.9–10 mmoL/L divided by the number of periods with at least one glucose value multiplied by 100. Time below range (glucose <3.9 mmoL/L) and time above range (TAR, >10 mmoL/L) were calculated similarly. Glycaemic variability was given as the coefficient of variation (%) of 15 min. Mean glucose values (standard deviation (SD)/mean glucose) multiplied by 100. Glucose monitoring indicator (GMI) was calculated as GMI (%) = 0.02392 × glucose (mg/dl) + 3.31 (GMI (mmol/mol) = 4.70587 × glucose (mmol/l) + 12.71).^13^

Collection of clinical data was approved by the local institution. No ethical approval was needed for this observational study.

### 2.1 Statistical analysis

Data that were normally distributed by visual inspection of Q‐Q plots are presented as mean ± SD, and paired data compared with Student's paired *t*‐test. Data for periods 1–4 were analysed by repeated measured one way ANOVA with calculation of SD between and within subjects. The 95% limits of agreement, that is, the normal distribution prediction interval for the difference between two measurements for the same individual, was calculated as within subject SD × 1.96 × √2. Data that were not normally distributed are given as median (interquartile range, IQR). Paired data were compared with Wilcoxon signed rank test and data from multiple periods by the Friedman test. The median of paired differences was estimated as Hodges–Lehmann median difference. TBR has a markedly skewed distribution. Log transformation of raw data was not possible because the tail of the distribution included approx. 3% with the value zero. To obtain a normal distribution, TBR was log transformed after omitting values below the 10 percentile level (corresponding to 0.61%) for pooled data from all four periods. Several other possible transformations of TBR data were considered including adding a constant 0.12% to zero values or omitting zero values. The different methods had no substantial influence on calculation of the within‐person SD. The statistical packages SPSS ver. 20.0 and R ver. 3.4.1 were used.

## 3 RESULTS

**FIGURE 1:**
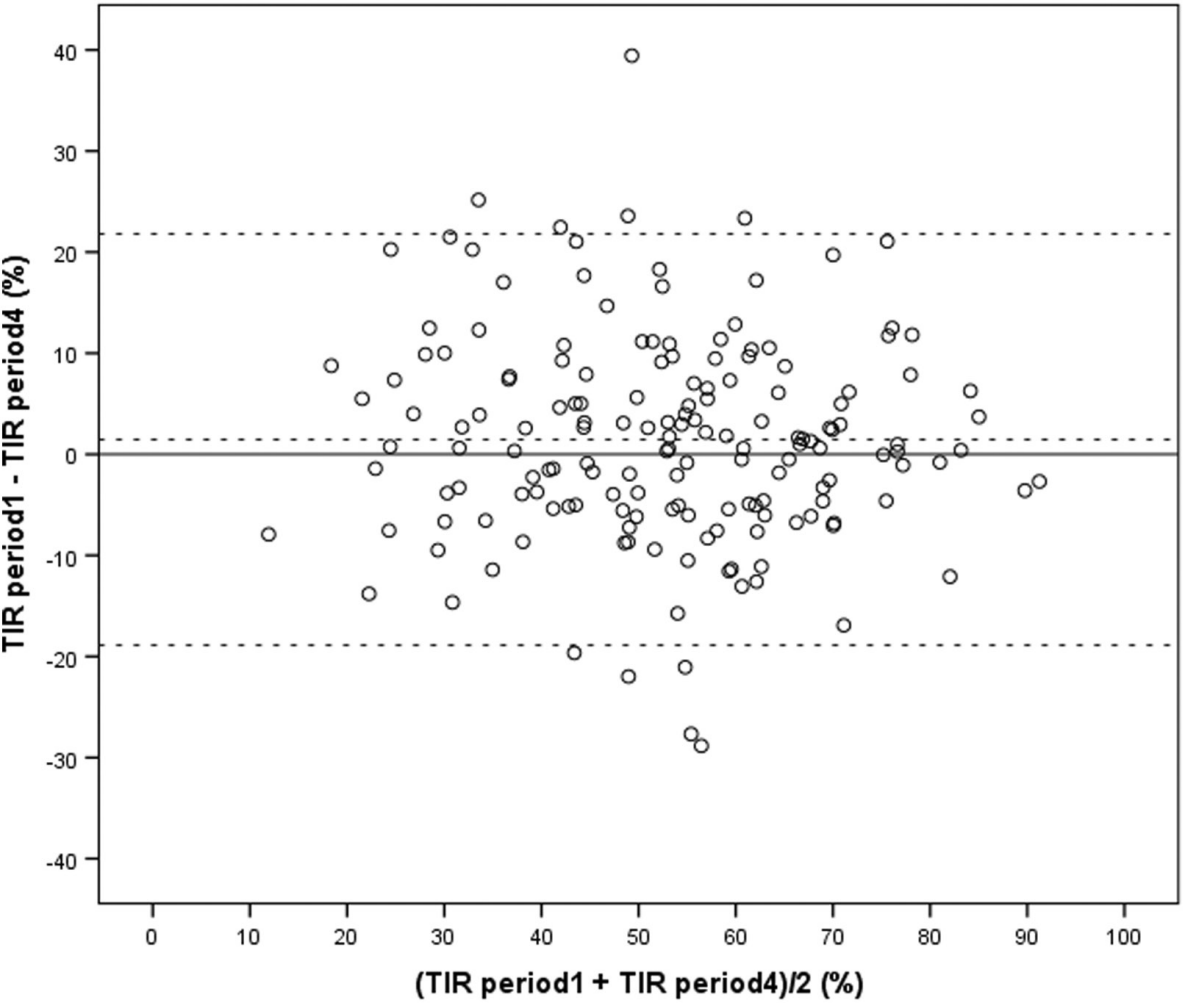
A Bland–Altman plot of the difference TIR period 1 minus TIR period 4 for 166 persons with type 1 diabetes plotted against their average. The dotted line denotes the mean difference 1.5% (SD 10.4%) and 95% prediction interval (Limits Of Agreement) from −18.9 to 21.8%. TIR is calculated from 2 weeks glucose data

Three of the 169 persons were excluded due to missing glucose values for one or two 14 days periods within the 8 weeks prior to download. Thus, data from 166 persons are presented. The mean age was 51.8 ± 14.2 years, mean diabetes duration 25.4 ± 14.3 years, and 93 (56%) were males. The majority (140 (84%)) used multiple daily injection (MDI), and the remaining 26 persons (16%) used continuous insulin infusion (CSII). Mean haemoglobin A1c was 7.6 ± 1.0% (59 ± 10 mmol/mol). The median number of scans for 30 days was 11 (CI: 9, 14.5) and for 90 days 11^8^, ^13^ (*n* = 161). Glycaemic metrics for each of the four periods of 14 days are shown in Table 1. No statistically significant difference between the four periods was noted. The difference between two TIR values (TIR period 1 – TIR period 4) separated by 1 month was 1.5% (95% CI: −0.1–3.1), *p* = .07, SD of the difference 10.4%. (Figure 1). The interval between two downloads for 61 persons was median 140 days (IQR 109, 203) and the difference in TIR (last—first measurement) was 1.7% (95% CI: −1.6–4.9) *p* = .31, SD of the difference 12.6% (Table 2).

**TABLE 1:** Glycaemic metric from 166 subjects calculated from four consecutive periods of 2 weeks

	Period 4 (weeks 7 + 8)	Period 3 (weeks 5 + 6)	Period 2 (weeks 3 + 4)	Period 1 (weeks 1 + 2)	Multivariate test	Between‐subjects SD	Within‐subject SD	95% limits of agreement for the difference between two measurements
TIR (%)	52.2 ± 17.1	53.1 ± 16.0	52.3 ± 15.7	53.7 ± 16.4	*p* = .11	15.02	6.34	± 17.6
TAR (%)	42.5 ± 19.0	41.3 ± 17.9	42.2 ± 17.6	40.8 ± 18.3	*p* = .11	16.79	7.05	± 19.6
TBR (%)	3.7 (1.6–7.4)	3.9 (2.0–7.7)	4.0 (1.8–7.6)	4.1 (1.8–7.3)	*p* = .94 (Friedmann)			
log(TBR) (%)	1.405 ± 0.865 (*n* = 150)	1.473 ± 0.849 (*n* = 152)	1.488 ± 0.847 (*n* = 148)	1.478 ± 0.869 (*n* = 148)	*p* = .51	0.677	0.487	± 1.349
e(log(TBR)) x/÷ tolerance factor	4.076 x/÷2.375	4.364 x/÷ 2.338	4.430 x/÷ 2.333	4.385 x/÷2.385		x/÷ 1.968	x/÷1.627	x/÷ 3.855
Glucose (mg/dl)	176 ± 35	174 ± 34	175 ± 34	174 ± 34	*p* = .31	32	13	± 36
(mmol/l)	9.8 ± 2.0	9.7 ± 1.9	9.7 ± 1.9	9.6 ± 1.9		1.8	0.7	± 2.0
GMI (%)	7.5 ± 0.8	7.5 ± 0.8	7.5 ± 0.8	7.5 ± 0.8	*p* = .31	0.8	0.3	± 0.9
(mmol/mol)	58.7 ± 9.3	58.2 ± 8.9	58.5 ± 8.8	58.1 ± 9.0		8.3	3.4	± 9.4
CV glucose (%)	38.2 ± 7.0	38.8 ± 7.0	38.5 ± 7.2	38.6 ± 6.6	*p* = .40	6.10	3.36	± 9.3
Active CGM time (%)	96.9 (94.1–98.5)	97.1 (93.1–98.2)	96.4 (93.2–98.2)	97.2 (94.1–98.5)	*p* = .07 (Friedmann)			

*Note*: Data are mean ± SD or median (IQR). TBR is log transformed data after winsorizing TBR <0.6% (corresponding to the 10th percentile level for all four periods) and presented both as mean ± SD and geometric mean +/÷ tolerance factor.

**TABLE 2:** Glycaemic metrics for 61 persons calculated from 2 weeks with an interval of more than 3 months

	First measurement	Second measurement	Second – first measurement	SD of the difference
TIR (%)	54.9 ± 15.1	56.5 ± 15.6	1.7 (*p* = .31)	12.6
TAR (%)	40.0 ± 16.0	38.3 ± 16.9	−1.6 (*p* = .34)	13.3
TBR (%)	3.9 (2.5–6.65)	2.8 (1.5–7.0)	−0.45 (*p* = .26)*	(−1.4, 0.45)^†^
log(TBR) (%)	1.477 ± 0.736 (*n* = 55)	1.264 ± 0.941 (*n* = 56)	−0.197 (*p* = .13) (*n* = 52)	0.922 (*n* = 52)
e(log(TBR)) (%) +/÷ tolerance factor	4.380 +/÷2.087	3.539 +/÷2.562	0.821 (Second/first)	+/÷1.022
Mean glucose (mg/dl)	170 ± 26	168 ± 29	2 (*p* = .51)	22
(mmol/l)	9.5 ± 1.5	9.4 ± 1.6	−0.1	1.2
CV %	37.6 ± 6.3	37.4 ± 7.7	−0.2 (*p* = .80)	5.9
GMI (mg/dl)	7.4 ± 0.6	7.3 ± 0.7	0 (*p* = .51)	0.5
(mmol/mol)	57.2 ± 6.9	56.7 ± 7.6	−0.5	5.7
HbA1c (%)	7.7 ± 1.0	7.5 ± 1.0	0.1 (*p* = .047)	0.5
(mmol/mol)	60.2 ± 10.5	58.9 ± 11.0	−1.3	5.0
Active CGM (%)	96.4 (94.1–98.7)	97.2 (94.8–98.3)	0.70 (*p* = .037)*	(0.05, 1.3)^†^

*Note*: Data are mean ± SD or median (IQR). TBR is log‐transformed data after TBR values <0.6% are winsorized and presented both as mean ± SD and geometric mean +/÷ tolerance factor.

^†^Hodges–Lehman median difference and (95%CI).

*The active CGM time is statistical significant longer on the second measurement than in the first measurement.

Within‐subject SD for TIR was 6.34%. It follows that the 95% prediction limit of TIR from 2 weeks is ±17.6% (=6.34 × 1.96 × $\sqrt{2}\;$). The risk that the difference between two TIR values by chance is larger than 10 percentage point is 26.4%, and the risk for a difference larger than 5% is 57.7%. If TIR is calculated from weeks 1–4 and weeks 5–8, the 95% prediction limits are ±13.1% (data not shown). The 95% prediction limits for mean glucose from 2 weeks are ±36 mg/dl (2.0 mmoL/L) and ± 0.9% (9.4 mmol/mol) for GMI (Table 1).

With the precautions needed due to winsorizing at the 10th percentile level of TBR, our results indicate that the limit of agreement for the ratio between two TBR values is between 3.86 and 0.26 (=1/3.86).

## 4 DISCUSSION

In clinical practice, a strong need exists to evaluate glycaemic interventions by assessing other metrics than HbA1c and to perform evaluation more frequently than can be expected to give rise to a change in HbA1c. The question is what order of magnitude of clinically relevant individual changes in TIR, mean glucose, and other glycaemic metrics can be considered within the range of normal variation. In our study, a change in TIR calculated from 2 weeks of ±17.6% is within the 95% limit of agreement. This vast variation should warn clinicians against over‐interpreting changes in TIR since it is difficult to draw firm conclusions by comparing a single pair of TIR values. Even if calculated from a four‐week period, the 95% prediction limit is high (±13.1%). The international consensus statement for interpretation of CGM data recognize that even a small (5%) increase in TIR is associated with a glycaemic benefit.^1^ A change in TIR of 10 percent point is considered clinically relevant for changes in retinopathy or albuminuria.^14^, ^15^ However, in the present study, a random change of more than 10 percentage point between two measurements of TIR is expected in more than 26.6% of the cases. In pregnancy, even a 5% change in TIR is clinically important.^16^

This study has some limitations. First, the result of isCGM was not blinded and TIR results cannot be considered truly spontaneous. The patients were expected to correct excursions in glucose and increase TIR, but changes in TIR were small and statistically insignificant. Another limitation is the data sampling frequency of 15 min. It is unknown if the higher sampling frequency in rtCGM (every 5 min) reduces variation of TIR. The most important limitation is the nature of the study of individuals with isCGM without alarms and only a minor proportion of patients with CSII and none with hybrid closed loop insulin delivery. Our results cannot be extrapolated to type 1 diabetes with other treatment modalities or isCGM used in other types of diabetes. We expect that the variation in TIR is lower in patients with insulin pumps with closed loop systems but to our knowledge, such data have not yet been presented. The software handling of CGM data from some companies allows presentation of two sets of TIR values based on either 1, 2, or 4 weeks. It is obvious from our results that comparison of ambulatory glucose profile reports should be interpreted cautiously.

The dimensioning of clinical studies to compare different technologies' impact on TIR depends on the SD of the difference between two measurements separated by a time period relevant for the study design. Previous studies have estimated a value of 12% with no specific references to the background data^17^, ^18^ or 14.5%^19^ referring to data on file.^20^, ^21^ We found a comparable value of 12.6% for TIR calculated for 2 weeks with an interval of several months and a lower value of 10.4% when comparing TIR calculated from 2 weeks separated by 1 month.

The strength of the study is a sizeable number of subjects included the fact that variation in TIR was estimated from glucose data derived from a normal daily life clinical setting in contrast to persons involved in a study, and the documented high active CGM time.

In conclusion, we have demonstrated a high variation of TIR in individuals mainly on MDI which should be taken into consideration when counselling persons with type 1 diabetes based on changes in TIR. More information about variation in TIR in persons treated with hybrid or advanced closed‐loop insulin delivery is needed.

## AUTHOR CONTRIBUTIONS

**Klavs Würgler Hansen:** Conceptualization (lead); data curation (equal); formal analysis (equal); funding acquisition (lead); investigation (lead); methodology (equal); project administration (equal); resources (lead); software (equal); supervision (equal); validation (lead); writing – original draft (lead). **Bo Martin Bibby:** Conceptualization (supporting); data curation (equal); formal analysis (equal); funding acquisition (supporting); investigation (equal); methodology (equal); project administration (supporting); resources (supporting); software (equal); supervision (equal); validation (equal); visualization (equal); writing – original draft (supporting).

## FUNDING INFORMATION

The study was financially supported by the Rosa and Asta Jensen Foundation.

## CONFLICT OF INTEREST

KWH has received honorarium as an advisory board member for Abbott Laboratories A/S, Denmark. BMB has no conflicts of interest.

## Data Availability

The data that support the findings of this study are available from the corresponding author upon reasonable request.
